# Environmental Triggers of Specific Subtypes of Agitation in People With Dementia: Observational Study

**DOI:** 10.2196/60274

**Published:** 2025-08-27

**Authors:** Hannah Davidoff, Alex Van Kraaij, Erika Lutin, Laura Van den Bulcke, Mathieu Vandenbulcke, Nick Van Helleputte, Maarten De Vos, Chris Van Hoof, Maarten Van Den Bossche

**Affiliations:** 1 ESAT, Department of Electrical Engineering, KU Leuven, Leuven, Belgium; 2 imec, Heverlee, Belgium; 3 OnePlanet Research Center, Wageningen, The Netherlands; 4 Geriatric Psychiatry, University Psychiatric Center KU Leuven, Herestraat 49, Leuven, 3000, Belgium, 32 016348000; 5 Neuropsychiatry, Department of Neurosciences, Leuven Brain Institute, KU Leuven, Leuven, Belgium

**Keywords:** dementia, agitation, multimodal sensing, digital health, medical informatics, observational study, quality of life, environmental factors, neuropsychiatric, mental illness, Alzheimer disease, experience sampling, linear mixed models, health informatics, clinical informatics

## Abstract

**Background:**

Among the most critical behavioral and psychological symptoms of dementia, agitation can lead to decreased quality of life of people with dementia and their caregivers. Monitoring triggers of agitation and its subtypes could enable early detection or prediction of agitated moments, which could be used to guide preventive or mitigating interventions. However, at this point in time, limited research exists on quantifying environmental triggers of agitation or its subtypes.

**Objective:**

In this paper, we aim to quantify the relationships between specific environmental factors and agitation as well as specific agitation subtypes, such as motor and verbal agitation.

**Methods:**

Using a cross-sectional repeated measures design, 37 people with dementia, admitted to a specialized neuropsychiatric ward for patients with dementia and severe behavioral and psychological problems, were each included for 1 week. During this period, the Pittsburgh Agitation Scale was filled in by the nurses on the ward following an experience sampling methodology to assess a patient’s agitation level on a momentary basis. Continuous environmental data (light, sound, and temperature) were collected from fixed sensors mounted on the ward. Generalized linear mixed models were used to quantify relationships between environmental variables and outcome variables (agitation, motor agitation, and verbal agitation). These models accounted for the hierarchical nature of our dataset as well as confounding factors, such as time of day and the room-level location of the patient. The time window for analysis was selected through a comparison of β coefficient estimates across various window lengths. Models were built up sequentially, per outcome variable, using selected features per environmental modality.

**Results:**

We found that different environmental factors captured in the window of 33 to 12 minutes before the agitation moment were most informative for different subtypes of agitation: mean light level (β=−0.61, 95% CI −1.12 to −0.10; *P*=.02) for motor agitation and SD of sound level (β=0.68, 95% CI 0.34‐1.02; *P*<.001) for verbal agitation. Contextual factors such as time of day (β range=0.51‐0.94; *P*<.05 to <.001) and room-level location (β range=0.85‐1.08; *P*<.01 to <.001) were also significant predictors of agitation.

**Conclusions:**

Integrating the key differences between predictors of verbal and motor agitation, respectively, the higher SD in sound level and the lower mean light level, in a model predicting the occurrence of subtype-specific agitation, could substantially improve model performance. Overall, these findings can aid in the development of predictive models for agitation based on environmental data and enable subsequent just-in-time interventions, improving the quality of life for both patients and caregivers.

## Introduction

Alongside the cognitive symptoms of dementia, behavioral and psychological symptoms (BPSDs) significantly impact the quality of life of patients and their caregivers [1-4]. Agitation, one of the most critical BPSDs, has been associated with increased caregiver burden [5], earlier institutionalization [6], and more rapid disease progression [7]. Behaviors that fall under the International Psychogeriatric Association’s definition of agitation include excessive motor activity, verbal aggression, or physical aggression [8]. The International Psychogeriatric Association further defines that for these behaviors to be considered agitation, they must be consistently or recurringly present for at least 2 weeks, have a large impact on interpersonal, social relationships or activities of daily living, and not be completely explainable by a comorbid condition. Several theoretical frameworks exist to explain the etiology of agitation in people with dementia. The progressively lowered stress threshold (PLST) model posits that behavioral symptoms, like agitation, arise when internal or external demands of the environment exceed a person’s stress threshold [9]. Additionally, this model hypothesizes that typical threshold levels are higher in the morning and lower throughout the day [10]. The PLST model is supported by Lawton and Simon’s [11] earlier environmental docility hypothesis, which states that people become more susceptible to environmental factors as their “level of competence” decreases.

Parallel to these frameworks, the Unmet Needs model [12] and the need-driven dementia-compromised behavior (NDB) model [13,14] focus on the needs of people with dementia and how they drive behaviors such as agitation. Both models stress the inability of a person with dementia to adequately communicate their needs. Unmet needs range from “loneliness or need for social interaction” and “boredom or sensory deprivation” to discomfort and pain [12]. One of the key factors in the Unmet Needs model is the need for adequate levels of stimulation [12] to mitigate boredom or social isolation in particular. Cohen-Mansfield et al [12] also state that different unmet needs relate to different behaviors. Specifically, they hypothesize that discomfort and pain would be associated with verbal agitation, while boredom or sensory deprivation would be associated with physically nonaggressive agitation [12]. Similarly, the NDB model postulates that different behaviors represent different unmet needs. For example, wandering could reflect the inability of a person with dementia to find their way or their need to avoid overstimulating or overcrowded locations. Vocalizations, on the other hand, could be an attempt to communicate a need. Algase et al [13] state that several background factors can affect NDBs: neurological factors, cognitive factors, and health status. In addition to these background factors, proximal factors such as emotions, physiological needs, functional status, and the social and physical environment can also impact NDBs [13].

These theoretical models form the inspiration for our research into what aspects of the environment could be triggers for the onset of agitation and how these triggers could differ by agitation type. Existing research has investigated univariate relationships between aspects of the environment and agitation. For example, Tartarini et al [15] have researched the relationship between temperature and agitation, where they found that the variation of temperature from “ideal” (22.6 °C) increased the frequency of agitation. Figueiro et al [16] have found that their bright light intervention decreased the occurrence of agitation. Janus et al [17] and Joosse [18] have looked at the impact of sound on agitation, determining that different aspects of sound (such as accumulation or variation) can increase agitation. However, these typically focus on an overall effect over a set period and not the effect of an environmental factor on a momentary level. In addition to unimodal research, there have been several studies looking into multimodal relationships between several environmental factors and agitation [19-22]. These studies point toward the relevance of analyzing environmental factors closer in time to the onset of agitation. A case study (n=1) by Au-Yeung et al [20] looked at environmental variables (ambient light, sound, humidity, etc) and other data from passive sensors using time windows of 8 hours aligned with the shifts of the nurses. There, the humidity and sound parameters were associated with agitation, although the humidity parameter was influenced by showers during visits from the included patients’ wives (which led to decreased levels of agitation). Homdee [21] used a sequence of nine 6-minute windows of environmental time series data forming a 54-minute input to a predictive model. HekmatiAthar et al [22] used windows of 30 minutes of environmental time series data to predict subsequent agitation. Both prediction modeling studies focused primarily on which machine learning framework performed best for this predictive problem and did not go into detail regarding feature importance. These analyses and modeling approaches also did not further refine models by agitation type, focusing only on agitation as a whole. Additionally, despite the clinical knowledge of increased agitation in the late afternoon or early evening [23-25], “sundowning,” these models do not take into account contextual factors like time of day. Here, we use an exploratory statistical approach to analyze important environmental factors not only on the presence or absence of agitation but also specifically on the presence or absence of motor and verbal agitation. This paper expands on current literature both through this extra level of detail on agitation and its subtypes as well as through the multimodal approach, providing a more holistic view on a patient’s environment.

The focus of this paper is to further understand which factors in the environment, measured by our study protocol [26], are associated with which type of agitation. With this, we aim to steer feature selection for agitation prediction models, eventually enabling just-in-time interventions as well as supporting the development of nonpharmacological alternatives (eg, environmental adjustment) to reduce agitation.

## Methods

### Patient Demographics and Study Design

The patients included in the study (N=37) were recruited from the population of a neuropsychiatric hospital ward specialized in high intensive care for people with dementia and accompanying severe BPSD with typically 4- to 8-week stays. The study was observational; no specific treatment interventions were introduced for research purposes, and routine care of patients continued throughout the study. The minimum inclusion criteria for the study were a diagnosis of dementia and the presence of agitation. Patients recruited from June 2021 to August 2023 formed a convenience sample. All patients admitted who met the minimum inclusion criteria or their representative were asked to participate. The clinical demographics of the included patients can be found in Table S1 in Multimedia Appendix 1. The ward unit layout consists of centralized communal living spaces with patient rooms and therapy rooms connected via hallways. More details can be found as additional information in Davidoff et al [26].

The study design from which the data used in these analyses originated was a cross-sectional repeated measures design, where patients were included for 1 week each. Sensor data, from both fixed and wearable sensors, were collected continuously throughout the week, and agitation labels were acquired by the nurses on the ward through the use of experience sampling [27] with a study-specific app, which prompted the nurse 9 times a day, semirandomly distributed. When prompted, nurses were asked to visit the patient and report the agitation level from that observation. On top of the 9 prompts, nurses had the option to self-initiate an ecological momentary assessment (EMA) to report an occurring agitation event and fill out the Pittsburgh Agitation Scale (PAS) after calming down the patient. More details related to the study protocol and rationale can be found in Davidoff et al [26].

### Ethical Considerations

The data collection protocol was approved by the ethics committee research of the University Hospitals Leuven (ID S62882). Since patients were experiencing dementia, written informed consent was mainly obtained through the patient’s representative. However, information on the study was explained as much as possible to the patient and on a level corresponding to the patient’s current cognitive capacities. Patient data were treated confidentially and pseudonymized. Participants did not receive financial compensation. The decision whether or not to participate in the study did not have any influence on the care or treatment patients received during their admission.

### Description of Data and Data Processing

The ground truth of the patient’s agitation state used in this study was the binarized score derived from the PAS [28], where the presence of each of the agitation subtypes (motor, verbal, resistance to care, and aggression) is scored on a scale of 0 to 4, and the presence or absence of each subtype is subsequently translated into 1 and 0, respectively. For this analysis, we focused only on verbal and motor agitation subtypes and not on the resistance to care and aggression subtypes, as these were rarely recorded as present in our survey dataset. This scale was a part of the survey notified for completion up to 9 times per day by the study-specific app.

Given the focus on the prediction and not the detection of agitation in this paper, a buffer (the spacing between the survey timestamp and the end of the window of environmental data analyzed) of 12 minutes was used. This was done to avoid detection of verbal agitation, one of the 2 most observed subtypes (as defined by the PAS) of agitation in our dataset, as verbal agitation could influence the measurement of sound on the ward. This buffer ensures that the input features are used to predict the subsequent onset of agitation and not detect agitation. Although we cannot completely exclude that verbal agitation had been ongoing beyond the buffer length, the buffer of 12 minutes is in line with buffers used in the literature [21]. Moreover, we have visually analyzed the effect of changing the buffer length and found that, as expected, the highest correlation between sound and verbal agitation is at the time of the EMA.

The length of the time windows explored in this paper ranged from 3 to 30 minutes at intervals of 3 minutes. This range is in line with time windows used in studies exploring the relationship between environmental parameters and agitation [21,22].

**Figure 1.**
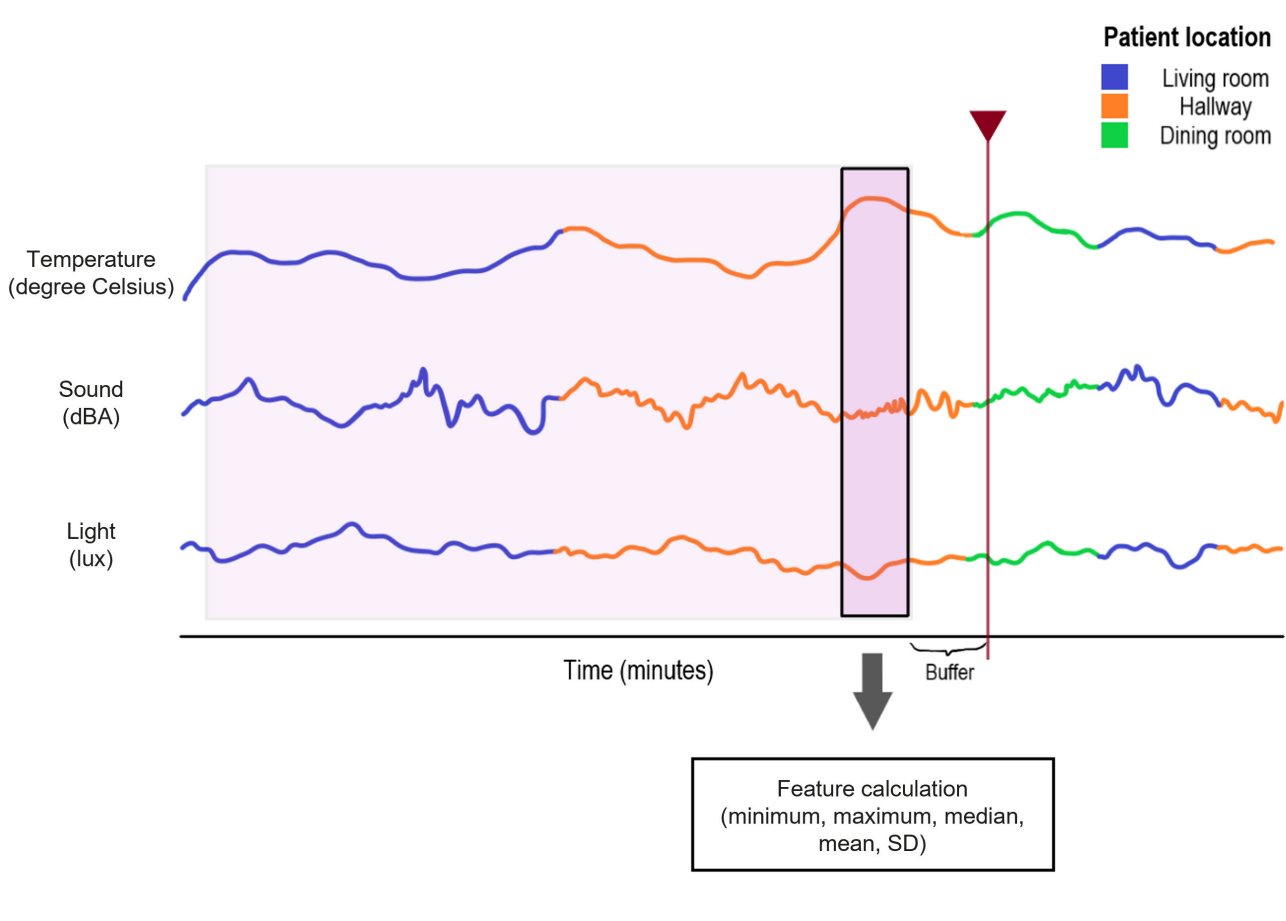
Data processing flow. (Not to scale) In this figure, 3 different standardized time series (one for each modality: temperature, sound, and light) are shown. The color of each time series denotes the location the data were extracted from. The red vertical line with a marker triangle indicates the timestamp of the survey. The spacing between this vertical line and the start of the shaded area is called the “buffer” and is used to avoid detection instead of the prediction of verbal agitation. It is set to 12 minutes. The small, shaded area is one example window. All the data for that window are used for feature calculation. For all other windows, the right side (end point) of the shaded area stays the same (−12 minutes from the survey timestamp), and the length of the time window is expanded to include more minutes of data before the survey timestamp. The final time window encompasses all the shaded areas. dBA: A-weighted decibel.

Using the survey timestamps, the environmental data were extracted for each of the 3 modalities focused on in this paper: sound (leqDBA), light (lux), and temperature (degrees Celsius). This was done by first determining the room the patient was in during the relevant time window. When an environmental sensor is present in that room, the time series segment includes the data from that sensor. If there was no environmental sensor, the data are left as not applicable, and the location is recorded as “off map” (eg, the patient location or environmental data for their location is unknown). Four rooms on the ward have 2 sensor enclosures present because of the above-average room area. When patient presence in any of those 4 rooms is detected, the respective sensors are averaged. If the patient visits multiple rooms in the relevant time window, the environmental data from each of the relevant sensors are appended. A moving average over 5 seconds is applied to each of the environmental data modality time series to smooth the effect of room transitions in the data. A visual representation of this data processing flow can be found in Figure 1.

Per the relevant time window, the room in which the most time was spent was recorded along with the exact amount of time spent in that room. Additionally, the number of data points present in the respective modality table was recorded per window. From all data points present per window, the following descriptive features were calculated: minimum, maximum, mean, median, and SD. Windows where any of the following quality conditions were not met were discarded from subsequent analysis: (1) the percentage of data points for which the patient location was unknown must be less than 50%, (2) there must be at least 50% of the expected number of data points present for that window length, (3) the majority room for that window must be a location with environmental sensors (eg, majority room is not “off map”), and (4) the survey was recorded within the time frame of 8 AM to 8 PM, as outlined in the protocol.

The first 3 criteria can be influenced by times when (1) the patient was not wearing the tracking wearable or (2) the patient is tracked to a location that does not have a direct environmental sensor present.

The features calculated per modality were standardized within the majority room, meaning that feature values are compared to all feature values for that specific location on the ward. A feature value of 1 would mean that the value of the feature is 1 SD higher than the mean value of that feature in that room. This makes the interpretation of the environmental sensor data generalizable across rooms and wards.

### Exploratory Modeling for Feature Selection

Given the hierarchical nature of this dataset (repeated measures within patients) and a binarized outcome variable, generalized linear mixed models (“glmer” models in the *lme4* package in R; R Foundation for Statistical Computing) were used for modeling with the family set to “binomial.” These types of models allow for random effects, taking into account group-level (in this case, patient-level) differences. The models developed in this step served as an initial exploration of informative features.

To explore the relationship between environmental variables and the different types of agitation, 3 different models (1 per outcome variable) were built per explanatory feature. The models follow the structure below.


$$\underset{\left[1\right]}{\text{Outcome variable}}∼\underset{\left[2\right]}{\text{explanatory variable}}+\underset{\left[3\right]}{\text{time\textbackslash{}\_group}}+\underset{\left[4\right]}{\left(1|\text{p\textbackslash{}\_id}\right)}$$

The outcome variable has 3 options: all agitation, motor agitation, and verbal agitation.

The explanatory variable has 15 values for iteration: the 5 standardized features (minimum, maximum, mean, median, and SD) for each of the 3 modalities (light, sound, and temperature).

The “time_group” stays constant throughout all models. This is the time of day split into 4 groups by the hour in which the survey was completed: (1) 8‐12 (exclusive), (2) 12‐16 (exclusive), (3) 16‐20 (exclusive), and (4) 20‐8 (exclusive).

p_id is the random intercept per patient. It is included to account for the nested structure of this dataset.

Per model built, we extracted the β coefficients quantifying the relationship between the explanatory variable [2] and the outcome variable [1]. These β coefficient estimates, analogous to “effect size,” can be interpreted as the log-odds change in the outcome variable with a 1 unit increase in the explanatory variable. This can be exponentiated to get the odds ratio. Since standardized features were used, a 1-unit increase is a 1 SD increase in the explanatory variable.

In addition to the feature-level analysis, different time window lengths ranging from 3 to 30 minutes were explored. An optimal window length was set for future modeling steps based on the window with the maximum amount of information (magnitude and significance of β coefficient estimates) across all features and modalities. The most informative features, in this window, were then selected per modality and outcome variable by the largest (absolute) β coefficient. These features were used as the environmental variable input in subsequent modeling steps.

### Modeling Agitation Types

Building on the selection of informative features in the initial exploratory modeling step, these models aimed to further quantify the relationships between environmental variables and agitation. This was done through a forward model selection process to build an optimal model per agitation type. Differences in informative features seen per outcome variable (ie, agitation type), both in magnitude and sign, were the reason for building an optimal model per outcome and comparing results. In this step, the selected features for light and sound were used as the basis for outcome-specific models. In addition to the time of day included in the exploratory modeling step, these models also included a majority room group to add additional context. The majority room group is a contextual variable that indicates the room where the majority of the time window was spent (see the Description of Data and Data Processing section) was grouped into 1 of 5 groups: “living room,” “dining room,” “hallway,” “patient room,” and “other.” This variable was included in the model to account for any confounding effects the patient’s location may have on the outcome. The temperature was dropped from the modeling analysis due to a lack of significant features for any outcome variable.

**Figure 2.**
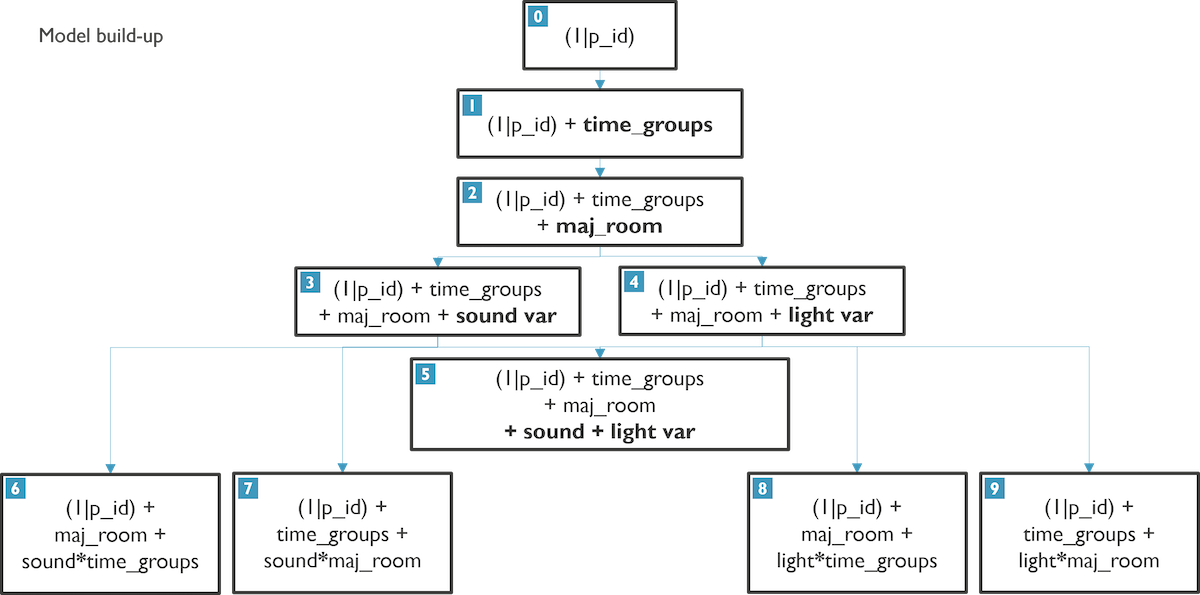
GLMER model build-up flow, which was used for each of the agitation types as outcome variables. The model improvement was checked at each step with ANOVA in R. This flow was used to find the most contributing environmental features.

For each of the outcome variables, the model was built starting from a model with only an intercept by the patient (model 0). The full model build-up flow along with each model structure can be found in Figure 2. First, the contextual variables are added one by one (models 1 and 2). Subsequently, both sound and light are added separately and then combined (models 3 and 4 and model 5). Finally, the interaction between either time or majority room (maj_room) was tested for both the sound-specific and light-specific models (sound: models 6 and 7 and light: models 8 and 9). The added value of including each variable versus the added complexity was tested with an ANOVA test using the *anova* function in R. The variable was kept in the model if the ANOVA test showed a significant improvement or if there was sufficient domain knowledge to support the inclusion of confounding variables (time or majority room). No Bonferroni correction or Benjamini-Hochberg correction has been used, as this analysis is purely explorative and not driven by a main hypothesis.

The coefficients were extracted for all model components along with model parameters for each of the models shown in Figure 2.

## Results

### Data Availability, Demographics, and Descriptive Statistics

Data on clinical demographics of the included patients (n=29) can be found in Table S1 of Multimedia Appendix 1 along with reasons and counts of patients not included in the analyses. Further details on the number of surveys present after each filtering step can be found in Table S2 in Multimedia Appendix 1. The filtering step that removes most surveys from the analysis is the “off map” filter.

**Figure 3.**
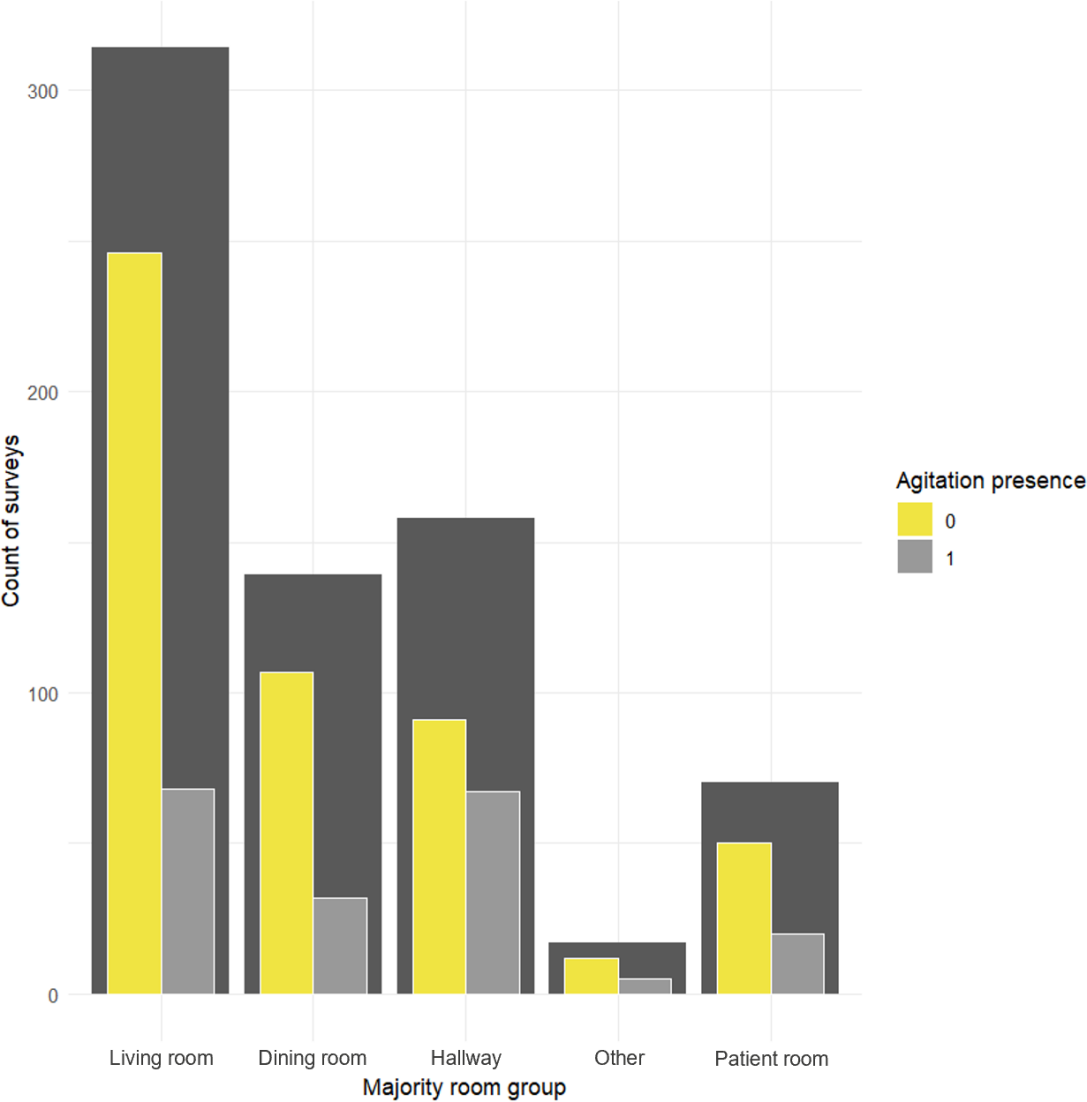
Occurrence and counts of surveys (dark gray), split for with annotated agitation (light gray) and without agitation (yellow) per room grouping.

Of the 694 surveys used in the mixed models, 503 are nonagitated. Of the remaining 191 recorded agitated surveys, 5 contain agitation that was neither motor nor verbal, 34 had only verbal agitation, 86 had only motor agitation, and 66 had coexisting motor and verbal agitation. The co-occurrence of motor and verbal agitation in this dataset is important in the subsequent interpretation of the results, specifically for verbal agitation where there are fewer “pure” verbal agitation surveys (n=34) than those co-occurring with motor agitation (n=66). For motor agitation, interpretation is still influenced by this co-occurrence but to a lesser extent due to the larger number of “pure” motor agitation surveys (n=86). Figure 3 shows the distribution of surveys and occurrences of agitation across the majority room group. The differing number of surveys recorded as well as the differing proportion of agitated surveys per room seen in Figure 3 aid in the interpretation of the room-level impact on subsequent model performance. A more detailed figure showing the proportion of agitation per room, split by type, can be found in Figure S1 in Multimedia Appendix 1.

### Exploring Optimal Time Window Length and Feature Selection

#### Time Window

The 21-minute window from 33 to 12 minutes prior to an EMA contained the majority of the maximum β coefficients, indicating that this window contained the most information for the prediction of agitation across modalities. For more details on the exploration of time window length, please see Figures S2 and S3 in Multimedia Appendix 1.

#### Feature Selection

**Figure 4.**
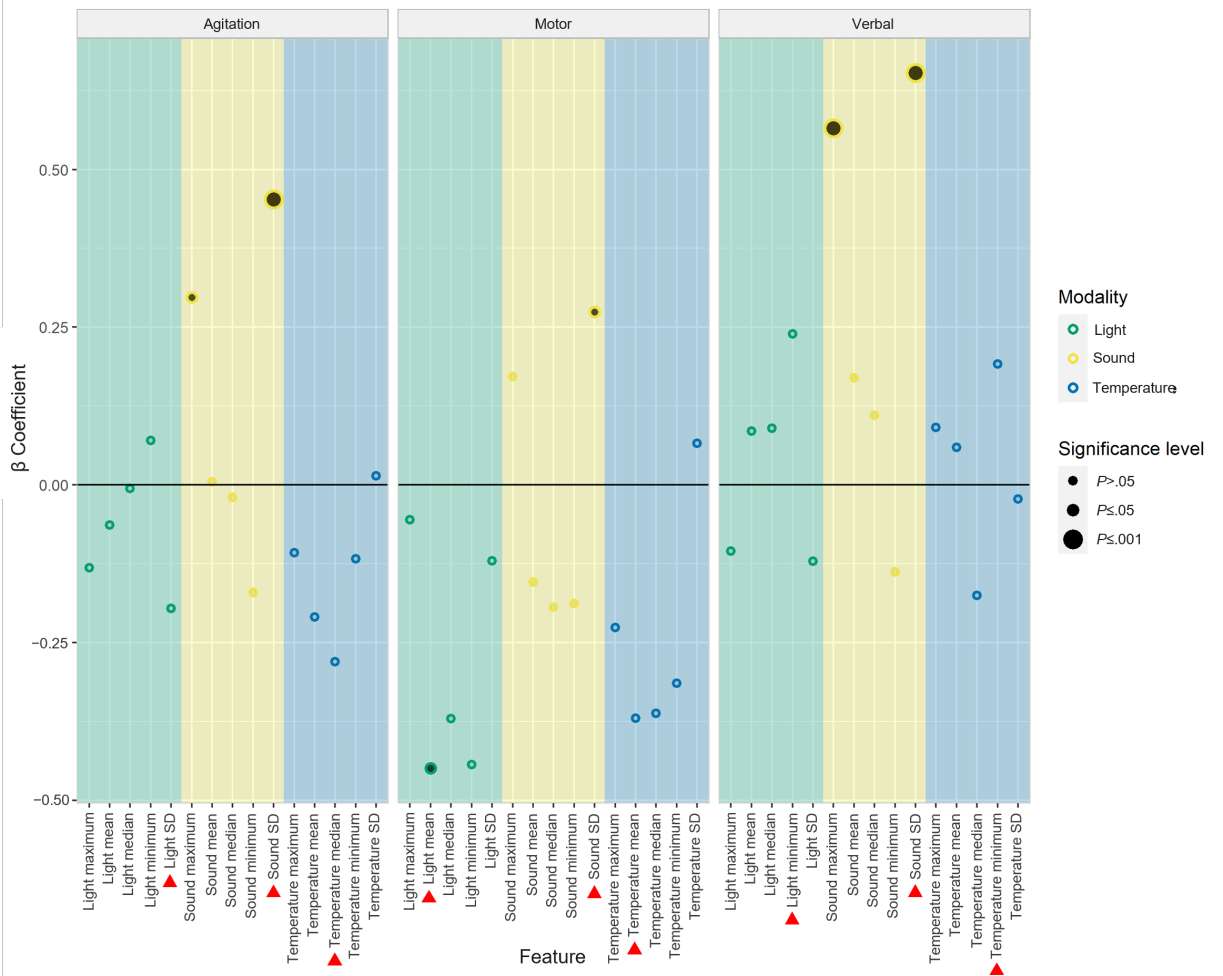
β Coefficients for all features of each environmental modality (light, sound, and temperature) per outcome variable (overall agitation, motor agitation, and verbal agitation). The data used as input for the features are derived from the period 12 to 33 minutes before the ecological momentary assessment. The features that were selected for further model build-up per modality and outcome variable are marked with a red triangle. The significance level is indicated with the size and filling of the dots.

For the selected 21-minute window, the β coefficients for each of the 5 features (minimum, maximum, median, mean, and SD) from each modality and for each outcome variable are summarized in Figure 4. To make agitation type–specific models, we extracted the most informative feature per modality for each outcome variable (agitation, motor agitation, or verbal agitation). The selected features are indicated with red triangles in Figure 4. Across each outcome variable, the SD of sound was selected for being the largest significant β coefficient (agitation: β=0.452; *P*<.001, motor: β=0.274; *P*=.04, and verbal: β=0.653; *P*<.001). The mean light level was only selected and significant for motor agitation (β=−0.449; *P*=.04). Other light parameters selected for having the largest β coefficients were SD (β=−0.196) and minimum (β=0.239) for agitation and verbal agitation, respectively. There were no features significantly associated with any of the outcome variables from the temperature modality. Median, mean, and minimum temperature had the largest β coefficients for agitation, motor agitation, and verbal agitation, respectively. Temperature was dropped from all subsequent modeling because of the lack of significant relationships between predictors and outcome variables at the preselected buffer and window lengths.

### Outcome-Specific Models

#### Model Optimization

The selected light and sound features were used to build up agitation type–specific models, following the build-up flow shown in Figure 2. For each variable or interaction added to the model, an ANOVA test was conducted to determine if the information added resulted in a significant improvement in the model fit. The results from these model fit tests can be found in Table 1.

**Table 1.** ANOVA model fit testing^a^.

Model^b^	Variable added	Agitation, *P* value	Motor, *P* value	Verbal, *P* value
0	Patient intercept only	N/A^c^	N/A	N/A
1	+ time_groups	.001	.008	.12
2	+ maj_room	.005	<.001	.02
3	+ sound parameter	.001	.08	<.001
4	+ light parameter (to 2)	.03	.004	.16
5	+ both light and sound (to 2)	<.001	.003	<.001
5	+ both light and sound (to 3)	.06	.003	.23
5	+ both light and sound (to 4)	.002	.06	<.001
6	+ sound*time	.26	.20	.16
7	+ sound*room	.74	.34	.93
8	+ light*time	.24	.47	.19
9	+ light*room	.24	.94	.17

a The *P* values here indicate the significance of the ANOVA test, which examines the added value of including each variable versus the added complexity of the addition. An insignificant result indicates that the added complexity of a model with an extra parameter is not worth the amount of extra variance explained by adding that parameter to the model.

b Model numbers correspond to the numbering in Figure 2.

c N/A: not applicable.

Each of the numbers of the models listed in column 1 of Table 1 corresponds to the model structures found in Figure 2. Including the “time_groups” variable in model 1 resulted in a significantly improved model fit for both agitation and motor agitation but not for verbal agitation. This indicates that the “time_groups” variable contributes important information for the prediction of agitation and motor agitation. This result, in combination with domain knowledge supporting the inclusion of time as a factor in agitation occurrence (eg, the PLST framework), led to the inclusion of “time_groups” in all subsequent models. In addition to time, including a second contextual variable “majority_room” also resulted in improved fits (Table 1). The inclusion of the selected environmental parameters had varying effects on model fits depending on the type of agitation used as the outcome variable. The addition of light led to significant improvements in models for agitation and its subtype motor agitation but not for verbal agitation. Conversely, the addition of sound led to significant improvements for agitation and its subtype verbal agitation but not for motor agitation. As seen in Table 1, including environmental parameters led to significant improvements in model fits compared to models where they were not included. However, including both environmental parameters in a single model was only informative when the most informative modality for that subtype was not yet included in the model. This is seen in the results when comparing model 5 to models 2‐4 per outcome variable; adding light when light was not yet included in a model for motor agitation resulted in a significant improvement in model fit. A similar trend was followed for the addition of sound for agitation or verbal agitation.

#### Model Parameter Estimates

The coefficient estimates for each of the predictor variables included in models 0‐5 can be found in Multimedia Appendix 1. The time group variables, for levels 2 and 3 (the hour groups 12‐16 exclusive and 16‐20 exclusive), were significant predictors of the occurrence of agitation and motor agitation. The β coefficients for time group 3 were higher (β=0.83‐0.94) than the coefficients for time group 2 (β=0.51‐0.61) across all agitation and motor agitation models. This indicates an increase in the log odds of agitation and motor agitation as the day progresses. The time group variables were not significant predictors of verbal agitation. The “hallway” level of the majority room group variable was a significant predictor for all types of agitation across all models (β=0.85‐1.08). The β coefficients for the “hallway” room group were consistently higher for motor agitation than for verbal agitation and agitation. The reference level used for the calculation of these estimates for the majority room group variable was the “living room.” This means that the effects of other locations are relative to the outcomes and variables at the living room locations.

Regarding the inclusion of environmental variables (models 3‐5), the SD of sound was a significant predictor of agitation and verbal agitation (β=0.42 and β=0.68, respectively). The β coefficient estimate for verbal agitation was higher than that of agitation. The mean light level was a significant predictor for motor agitation only with a coefficient estimate of −0.61 for the light-only model (model 4) and an estimate of −0.60 for the light and sound model (model 5). These negative coefficient estimates indicate that an increase in the mean light level would lead to a decrease in the log odds of the outcome variable (motor agitation).

## Discussion

### Principal Findings and Comparison to Prior Work

The results presented in this paper explore the relationship between environmental factors, such as light, sound, and temperature, on agitation and 2 of its subtypes: verbal and motor agitation. Exploratory models were used to identify the most informative features per modality and outcome variable, based on the magnitude and significance of their relationships to agitation, motor, and verbal agitation. With these features identified, agitation and agitation type–specific models were built up, considering additional contextual variables like the room-level location of the patient, to quantify further the relationship between each selected environmental variable and the relevant outcome variable.

With the time window length set at 21 minutes, the most informative features were selected using the results summarized in Figure 4. The selected features were then used in agitation type–specific models built up from a model containing only a random intercept per patient (model 0). The most informative features were not consistent across all modalities and outcome variables, already an initial indication of possible type-specific triggers of agitation. This indication is reinforced throughout the forward selection process, where the most informative modality also differed depending on the outcome variable analyzed.

For motor agitation, light (mean) was consistently the predictor with the largest β coefficient estimate. This is present in Multimedia Appendix 1 (β mean light=−0.61; *P*=.02) as well as in the model build-up results summarized in Table 1. After the inclusion of both contextual variables, time of day and majority room, the addition of light led to an improved model fit for motor agitation (*P*=.004), where the addition of the SD of sound did not. This further solidified the specific importance of light for this type of agitation. Current literature relates indoor light levels to spatial or temporal disorientation [29,30], where lower light levels lead to increased levels of disorientation, which can manifest as wandering. Additionally, light and sundowning are thought to be related through the disturbed sleep-wake cycle often seen in people with dementia [16,23]. While studies on the impact of lighting in BPSDs have shown mixed results, several have shown an association between lighting parameters and specific behavioral symptoms. Figueiro et al [16] reported that their lighting intervention (consisting of tailored “bluish-white” lighting) significantly decreased agitation scores on the Cohen-Mansfield Agitation Inventory and resulted in an increase in circadian entrainment. The increase in the β coefficient estimates when comparing time group 2 and time group 3 (2: β=0.51‐0.61 and 3: β=0.83‐0.94) could also be explained by a relationship between motor agitation and sundowning. This contrasts with the insignificant time predictors seen in the models for verbal agitation. It is possible that observed sundowning is primarily driven by the observation of motor agitation. Algase et al [31] reported that wanderers wandered less when light levels were lowered. At first glance, this contradicts the result found here; however, this could be explained by a difference in baseline light level [31]. Their study is based on the NDB model, which identifies internal or “background” and proximal factors for behaviors like wandering and agitation. However, in this model, and in other theoretical frameworks focusing on agitation triggers, there are often 2 sides to environmental triggers: overstimulation and understimulation or boredom [12,32]. In this case, our result showing that low mean light levels increase the occurrence of motor agitation, and the result of Algase et al [31] showing that lowered light levels decreased the occurrence of wandering (a subtype of motor agitation), can coexist. This is especially the case when comparing results with different baselines: the light at one ward can be baseline overstimulating, therefore leading to decreases in wandering when stimulation is lowered, while another ward—like the one here (median light levels per room <100 lux; median 22, IQR 7-64 lux)—can be baseline understimulating (eg, low light levels) and lead to decreases in wandering or motor agitation when stimulation is increased. Incorporating additional information on light parameters (eg, circadian stimulus of light, correlated color temperature, and red, green, and blue) on the ward could expand this analysis, given the relationship between the specific aspects of light and the regulation of the circadian system [16,33]. It is also possible that differences between our results and those from Algase et al [31] originate from subtle differences in wandering and motor agitation. Despite the disagreement on the influence of light on agitation, the significance of the “hallway” room location as a predictor variable for agitation (β=0.85‐1.08 depending on the model structure) is in agreement with the location, where the highest proportion of wandering (along with in the dining room) is seen in Algase et al [31].

For verbal agitation, sound was the most informative modality. This was seen both in the model build-up ANOVA results (Table 1) and in the predictor estimates from the models in Table S3 in Multimedia Appendix 1. The SD of sound was chosen as the most informative sound feature across all outcome variables, with the largest β estimate (β=0.653) for verbal agitation, followed by agitation (β=0.452), and 0.274 for motor agitation. This result is in line with Algase et al [31], where wandering was reported to occur more frequently with higher SDs of sound. However, wandering is considered a subtype of motor agitation [28]. Therefore, we would expect that the SD of sound is also a significant predictor of motor agitation. For our results, this is not the case, although the coefficient estimate does have the same sign, indicating a similar trend (increased SD of sound, increased motor agitation). The difference in magnitudes of the β coefficient estimates between SD of sound and verbal agitation compared to agitation is likely driven by the “agitation” outcome variable, including all types of agitation, and not specifically comparing the presence or absence of verbal agitation. Sound, in general, being a significant predictor for agitation, is in line with prior research, showing that increased sound levels lead to increased agitation [17,18]. Following the NDB model, this could be influenced by the number of people present in a particular room (eg, possible crowding) increasing sound parameters [13]. The relationship between verbal agitation and sound parameters could also be due to overstimulation, as hypothesized in the Unmet Needs model [12].

Temperature was left out of model optimization, as it was not significantly related to any outcome variables. Existing research has shown that the variation in temperature from ideal is a predictor of agitation. It is therefore possible that this variation, which could be in either direction from ideal (too hot or too cold), is not adequately captured in the features used for these analyses. Future work could use different features to quantify, for example, the amount of time that the temperature differed from the mean by at least 1 or 2 SDs. This feature is more closely aligned with the significant predictor reported in Tartarini et al [15] than the SD of temperature used here.

These results, correlating environmental sound parameters in time windows before an agitated survey is recorded, build upon existing research of predictive agitation models by making the distinction between different agitation types and possible environmental triggers. This is in line with the Cohen-Mansfield “Unmet Needs” model [12], where different unmet needs (possibly triggered by environmental parameters) are said to be related to different behaviors, like the expression of agitation of different types. Key differences between predictors for motor agitation and verbal agitation have been highlighted, and taking these differences into account could substantially improve the performance of predictive models. These agitation type–specific results have the potential to drive explainable machine learning models by keeping clinically interpretable knowledge in the model development process. Nevertheless, if an agitation model is being developed without the integration of type-specific information, sound appears to be a stronger predictor due to improved model fits (Table 1) compared to the addition of light as well as larger coefficient estimates across models as seen in Table S3 in Multimedia Appendix 1. It is also important to keep important contextual information about the agitation episode in the model as well, as indicated by the significant time of day and majority room parameters. This is in line with the work by Stojchevska et al [34], where adding context to stress modeling improved model performance.

### Strengths and Limitations

A larger sample size, allowing for a confirmatory analysis instead of an exploratory one, could further strengthen the results reported here. It is possible that β coefficients not reported as significant would be significant when enough surveys were included to capture the full range of each feature more confidently. Additionally, the dataset size here could explain disagreements with existing literature. Other research has investigated how specific aspects of sound were correlated with agitation. Including frequency-level features of sound could allow for a more in-depth analysis of the relationship between sound and different agitation types.

### Future Directions

Future work can combine the environmental triggers identified here with the quantified physiological response seen when a patient is agitated to form a more holistic model of agitation without the need for survey-based labels. Furthermore, due to the differences seen between agitation subtypes, future work comparing the efficacy of interventions for specific types of agitation may be more informative than focusing on all types of agitation.

### Conclusions

The results presented here provide fundamental information supporting the understanding of environmental triggers of agitation. Using various theoretical frameworks to guide this analysis, we have quantified significant relationships between several environmental factors and specific agitation types as well as agitation as a whole. We found that motor agitation and verbal agitation differ in the environmental data modality that provides the most information.

Motor agitation was shown to be most related to light levels on the ward, where low light levels increased the levels of motor agitation shown. In contrast, increased verbal agitation was related to higher SDs of sound levels. These results are in line with existing theoretical frameworks of the etiology of agitation, particularly they focus on balancing stimulation levels on the ward. In addition, we have identified the relevant time window for (just-in-time) prediction of agitation using these factors. Future agitation prediction models can incorporate this additional domain knowledge of agitation type–specific triggers and related contextual parameters to improve model performance. Furthermore, nonpharmacological interventions aimed at the patient’s direct environment can be tailored depending on the type of agitation most often expressed. Models like these, supporting autonomous identification and prediction of agitation, can help alleviate the burden on the caregiver by reducing the impact of agitation on care, therefore improving both patient and caregiver quality of life.

## Supplementary material

10.2196/60274 Multimedia Appendix 1 Further study and analysis details.

## Abbreviations

BPSD: behavioral and psychological symptom
EMA: ecological momentary assessment
NDB: need-driven dementia-compromised behavior
PAS: Pittsburgh Agitation Scale
PLST: progressively lowered stress threshold
