# Supplementary material for: Environmental Triggers of Specific Subtypes of Agitation in People With Dementia: Observational Study
Source: JMIR Form Res. 2025 Aug 27;9:e60274. doi: 10.2196/60274 (PMC12384682; doi:10.2196/60274)
Supplement: Multimedia Appendix 1 [file formative-v9-e60274-s001.docx]

**Table S1.** Demographics of included patients in the study (mean + std.s reported). Not included in these demographics: Incomplete inclusion: n=2, no agitation observed during study week: n=1, technical failure/limited wearable compliance: n=5.

| **Sex** (#) | **Age** (Years, mean + std.s) | **Diagnosis** (#) | **Time since diagnosis** (Years, nan: 2) | **MMSE**  (nan: 2) | **CMAI** | **NPI**  (FxS) | **Cornell** |
| --- | --- | --- | --- | --- | --- | --- | --- |
| 11 F  18 M | 79.45±7.45 | *Alzheimer’s Disease*: 10  *Vascular*: 3  *Mixed (AD-VaD):* 4  *With Lewy Bodies:* 4  *Multiple Etiologies:* 3  *Other (due to another medical condition):* 1  *Not otherwise specified:* 4 | 3.02±1.79 | 12.93±6.39 | 55.62±15.06 | 32.24±16.07 | 9.41±4.30 |
| MMSE – Mini Mental State Exam, CMAI – Cohen Mansfield Agitation Inventory, NPI – Neuropsychatric Inventory, FxS – Frequency & Severity, F/M – Female/Male, AD-VaD – Alzheimer’s Disease & Vascular Dementia. | | | | | | | |

**Table S2.** Number of surveys present after each filtering step explained in ‘Description of data and data processing’. Split by modality. The number used in the combined modality models is **across** modalities (n=694) and can be explained by inconsistent data quality across modalities.

| **# of surveys: 1193** | **Sound** | **Light** | **Temperature** |
| --- | --- | --- | --- |
| **Criteria filtered out** |  |  |  |
| Location unknown >= 50% | 750 | 748 | 739 |
| Proportion present < 50% | 719 | 716 | 713 |
| Time group ==4 | 717 | 714 | 711 |
| Majority of window (present) in unknown location | 698 | 697 | 695 |

**Table S3.** Parameter estimates by model in Figure 1: Model build-up flow. Split by outcome variable. P<.001 = ***, P<.01 = **, P<.05 = *

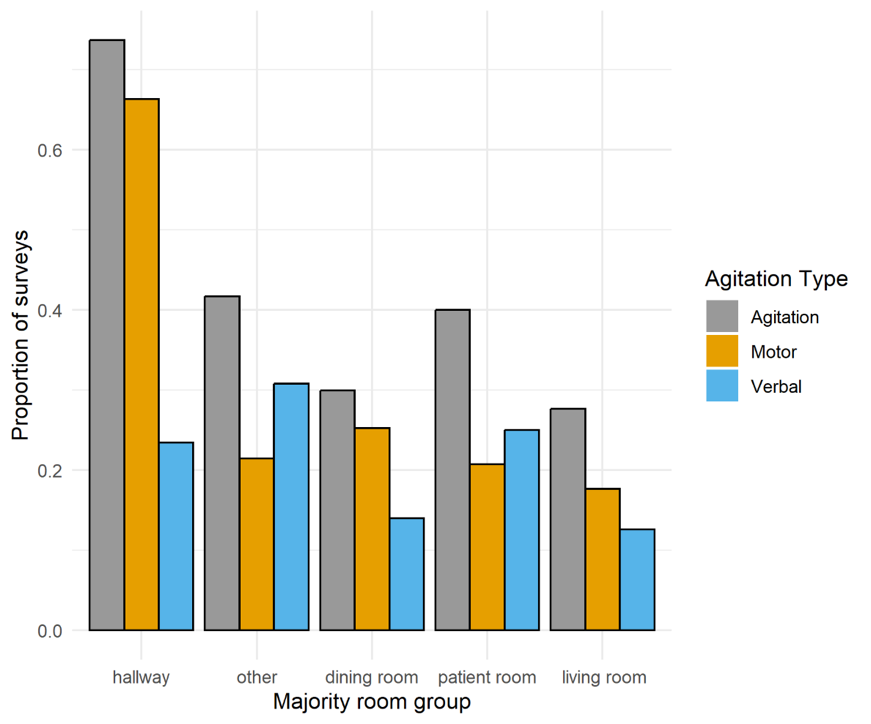


**Figure S1.** A more detailed figure showing the proportion of agitation per room, split by type.

**
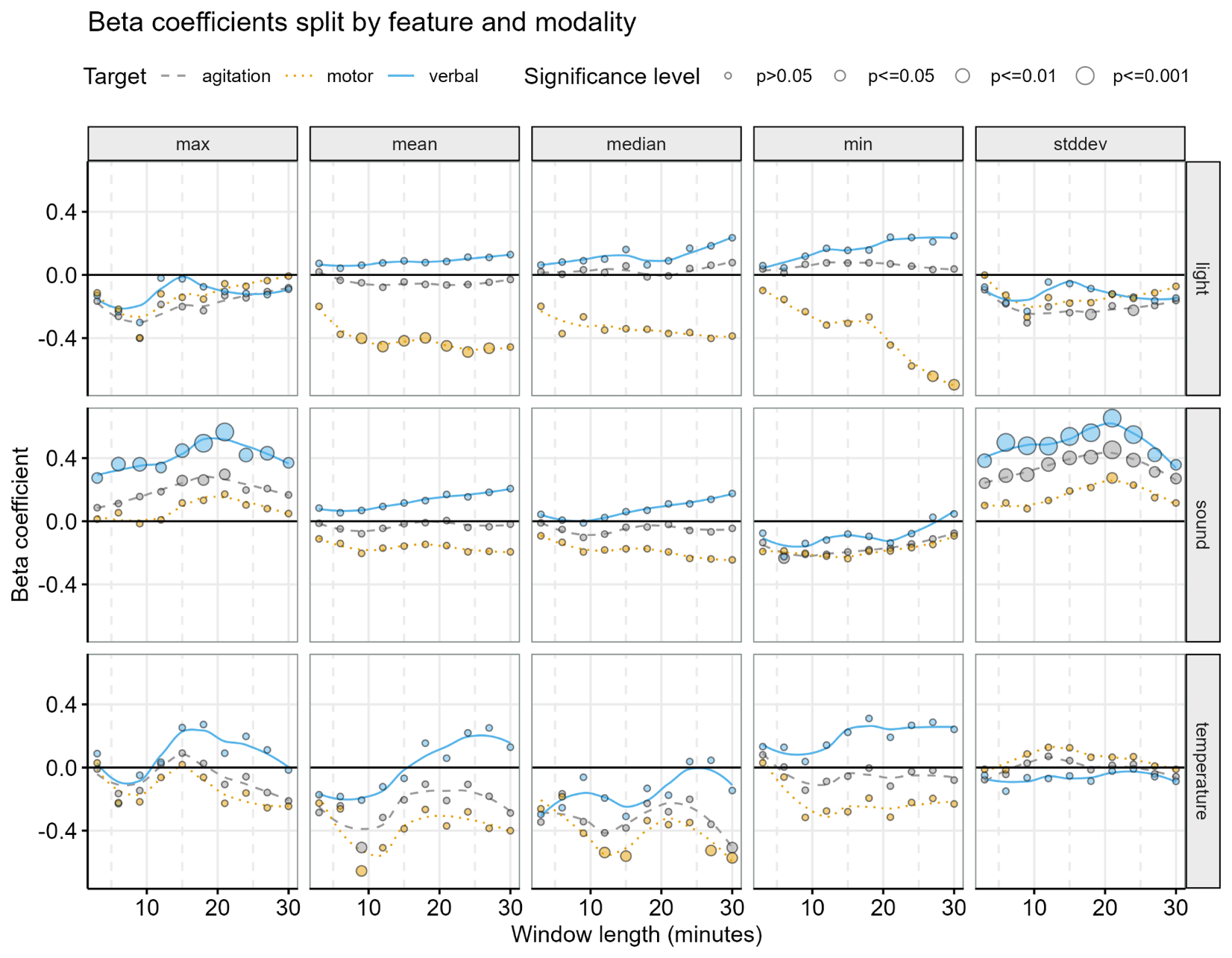
****Figure S2.** The beta coefficient estimates per time window length explored for each of the five feature variables across each modality (light, sound, and temperature) and outcome variable (agitation, motor agitation and verbal agitation), colored and line type by outcome variable. Grey dashed: agitation. Yellow dotted: motor agitation. Blue solid: verbal agitation. The size of each data point is determined by the significance level of the beta coefficient estimate, where the smallest size circle is an insignificant coefficient. In the first row, the mean light level had several significant beta coefficients across different time windows for motor agitation only. These coefficients were consistently negative (and significant at the P<.05 level: [.023 - .047] from window length of 9 – 27 minutes) indicating that low light levels over a period are related to an increase in the occurrence of motor agitation. In the second row, focusing on sound features, both the maximum and standard deviation of sound show consistent significant beta coefficients (P-values ranging from <.001-.04) across all time windows for verbal agitation. For both features, the coefficients rose to a peak level at the 21-minute window length and subsequently dropped off. Additionally, the standard deviation of sound was a significant predictor of agitation (P-values ranging from <.001-.04) in all window lengths. For motor agitation, the standard deviation of sound was only a significant predictor in the 21-minute window length (P=.04). In the third row, focusing on temperature features, there were no obvious patterns for the sporadic significant beta coefficients shown.


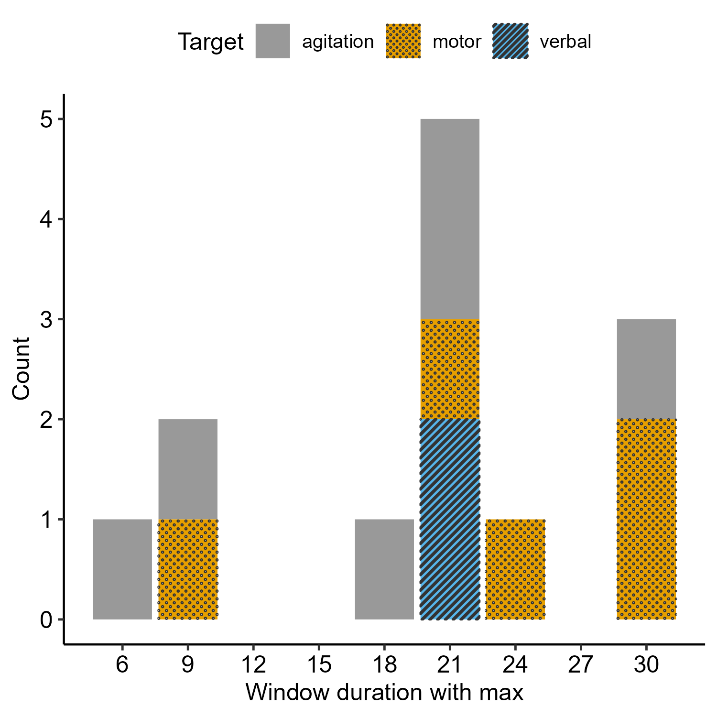


**Figure S3.** Count of maximum value of scoring parameter by window-length. The window length in which the maximum values of the scoring parameter (factorized significance level * beta coefficient, where insignificant values are multiplied by 0) were found, is 21 minutes.
